# Dynamic Cost-Aware Routing of Web Requests

**DOI:** 10.3390/fi10070057

**Published:** 2018-06-21

**Authors:** Gandhimathi Velusamy, Ricardo Lent

**Affiliations:** 1 Computer Science, University of Houston, Houston, TX 77204, USA; 2 Engineering Technology, University of Houston, Houston, TX 77204, USA

**Keywords:** autonomous systems, learning automata, energy, web, datacenter, QoS

## Abstract

Work within next generation networks considers additional network convergence possibilities and the integration of new services to the web. This trend responds to the ongoing growth of end-user demand for services that can be delivered anytime, anywhere, on any web-capable device, and of traffic generated by new applications, e.g., the Internet of Things. To support the massive traffic generated by the enormous user base and number of devices with reliability and high quality, web services run from redundant servers. As new servers need to be regularly deployed at different geographical locations, energy costs have become a source of major concern for operators. We propose a cost aware method for routing web requests across replicated and distributed servers that can exploit the spatial and temporal variations of both electricity prices and the server network. The method relies on a learning automaton that makes per-request decisions, which can be computed much faster than regular global optimization methods. Using simulation and testbed measurements, we show the cost reductions that are achievable with minimal impact on performance compared to standard web routing algorithms.

## 1. Introduction

The Internet traffic continues increasing driven by multiple factors, including the proliferation of web of things [1], popularization of wearable computers, business globalization, and new technologies for machine-to-machine communications, high speed network access, and content delivery. With the rise of affordable personal gadgets like smart phones, the number of network devices and connections became 2.3 per-capita in 2016 and it is estimated that it will reach 3.5 per-capita in 2021, with 58% of the global population gaining access to the Internet [2]. Despite the benefits, the growth of the network increases global energy consumption and brings many side-effects, such as green house emissions. Datacenters (DC) have become a mission critical computing infrastructure for the Information Communication Technology (ICT) sector. It has been estimated that the energy that will be consumed by DCs in the United States by 2020 will be 140 billion kWh (kilowatts-hour), which is equivalent to the power generated by 50 power plants. This amount of energy will cause 150 tones of carbon pollution [3]. With varying electricity prices, DC operators seek to reduce their total cost of ownership to increase the return on investment.

In the U.S., the wholesale electricity market is administered by Regional Transmission Organizations (RTO) and Independent System Operators (ISO) across 10 regions. Electricity price rates are spatially different across U.S. electricity markets and are decided based on the generation methods, availability, demand, and transmission costs over the grid. The RTO/ISO administers the electricity generation and transmission over the grid. Normally, electricity that is generated with low-cost generation methods, e.g., using coal or nuclear power, results in low prices. However, when demand is high, or increase rapidly, the extra demand needs to be fulfilled using higher cost generation methods, natural gas, that results in higher prices [4,5]. These features make electricity prices to vary greatly among not only across different regions, but also with time.

In the web industry, service delay is a prime quality factor. Web requests must receive service with the quality-of-service (QoS) level stated in the service level objective. This is only possible through the use of redundant server clusters, to provide proper availability, scalability, and fault tolerance. For example, content servers are commonly replicated at the edge of the network—to be as close as possible to the end-clients. Servers need to be replicated across geographical locations to cater to more clients and to reduce service delay. This deployment strategy allows operators to better deal with the unpredictable nature of the web traffic. A server consumes a minimal amount of energy when it is powered on, but its energy demand increases proportionally to its workload. Response times also increase along with the supplied load. It is possible to regulate to some extent the electricity costs of an organization that operates DCs at different regions through web traffic routing. However, it is most important to preserve the desired QoS in this operation as the network state and current servers’ load will affect response times. Therefore, there is a potential tradeoff between response times and the energy consumption involved in routing web requests to different DCs. The solution to the problem is not trivial as the network state is normally unpredictable. Both the delay involved in a network state collection from distributed servers and the computing overhead prevent the use of a high frequency optimization of web routing by convex optimization or linear methods. We propose a solution based on learning automata—the Cost Aware S-model Reward Penalty Epsilon (CA-S) method seeks to reduce the average cost in serving web requests with replicated web servers deployed on different geographical regions. We formulate a cost objective that includes energy costs and revenue loss. The latter factor captures the impact that high delay can cause to users and the operator.

The learning automaton makes routing decisions for each incoming request by assessing response times and energy prices at the different server locations through action selection probabilities. Then, it probabilistically forwards the request to the server that minimizes the cost. Our contributions are:(i) a learning automata-based method for routing web requests considering both the differences in energy prices across locations and the revenue loss potentially produced by high response times; (ii) a simulation study of the performance impact of learning parameters, and; (iii) an implementation of the method for Apache Traffic Server and testbed evaluation in CloudLab [6]. We have compared our results to common baseline methods: minimum cost flow algorithm and round robin algorithm. Our experimental results show that the total average cost of serving web responses could be reduced up to 33% when compared to the minimum cost flow dynamic server selection algorithm and up to 49.2% compared to the common round robin method.

## 2. Literature Review

The problem of datacenter energy optimization has received good attention in the literature. We review representative works with a focus on energy optimization through request or workload re-routing with energy consideration.

### 2.1. Load Balancing by Spatial Pricing

The U.S. electricity market has been analyzed in detail with prices from 30 locations [7] and it has been proved that 40% savings are possible with dynamic price-aware routing through a simulation study using Akamai’s content delivery network (CDN) traffic traces. A two-level technique to reduce the electricity cost in multi location DC has been proposed [8]. The spatial variations in electricity prices were exploited at the first level and scheduling strategies were proposed at the second level to distribute the requests among heterogeneous servers available at the selected DC.

The stratus system proposed in [9] used a Voronoi graph partition algorithm to select a DC for servicing the requests. The service time, cost of the energy consumption and carbon emission were considered as metrics to be optimized in selecting a DC. A distributed cloud framework to reduce power consumption in the cloud based on dynamic speed scaling of processors was proposed [10]. Temporal and spatial variations in electricity prices were exploited to balance the load across the clusters and to reduce the energy consumption.

The total electricity cost reduction is formulated as a constrained mixed-integer problem and solved using Brenner’s polynomial algorithm [11] with a simulation study using prices from three locations of Google Internet datacenters. The energy consumption was optimized using a global load balancing policy and at a server level management by switching *on/off* using a mathematical analysis study [12]. A corrective marginal cost algorithm was proposed to solve the total energy cost by using a nonlinear integer programming optimization model [13]. A Big Data analytic framework was proposed to minimize the total energy cost and delay for running big data analytic jobs on geographically distributed big data sets using a stochastic optimization method [14]. A recent work [15], proposes a solution to the cloud service providers who procure electricity from deregulated electricity market to optimize total electricity cost and bandwidth cost by dynamically routing the requests to DCs where electricity is cheaper.

### 2.2. Routing Work Loads to Renewable Energy Powered DCs

A global load balancing of web requests to DCs where the renewable energy is maximum or the energy is cheaper was proposed [16]. Renewable energy can be stored using uninterrupted power supplies (UPS) to be later used during power fluctuations to reduce energy costs. The power stored in UPS can be also used to reduce the peak power demand cost—known as peak shaving during the peak utilization hours [17]. Two algorithms were proposed [18] to reduce the use of brown energy to zero by shifting workloads to the DCs in regions where the energy cost is low and by switching off servers at lightly loaded DCs. The receding horizon control (RHC) algorithm for homogeneous settings and the averaging fixed horizon control algorithm (AFHC) for heterogeneous settings were proposed.

A fuzzy logic-based load balancing method was proposed to route the workload among geographically distributed datacenters to reduce electricity costs by considering renewable energy availability, brown energy consumption and average electricity cost at DC locations [19]. A reactive global load balancing and auto scaling of servers were proposed to reduce electricity cost and brown energy usage by routing requests to sites with higher renewable energy and lower costs [20]. A cost function that comprises a linear combination of energy cost and lost revenue due to SLA violation is used to route the job requests. A distributed geographical load balancer was proposed [21], in which the jobs are routed to DCs using “follow the renewable routing” policy to DCs where the proportion of brown energy to total energy production is at a minimum.

A two-time-scale load balancing algorithm, TLB-ARMA, was proposed to route web requests to a DC where the electricity price is minimum and powered by wind energy [22]. The air temperature was measured at the beginning of scheduling intervals to compute cooling efficiency and to predict the wind power. A constant number of servers were maintained to avoid power wastage in frequent *on/off* of servers. The most recent work [23] proposes a renewable aware load balancing policy using a combination of Reinforcement Learning and Neural Networks to optimize Big Data Analytic jobs across geographically distributed datacenters.

An energy-aware pricing in the three-tiered cloud service market was proposed [24] to minimize the total energy cost of the system by promoting integration of renewable energy in the system. The square root staffing law policy was used to maintain required number of servers in the *on* state to provide better QoS and to increase the revenue to the Infrastructure-as-a-Service (IaaS) providers. A Global load balancing policy was used to distribute workloads of software-as-a-service providers (SaaS) to IaaS providers across spatially different electricity markets while reducing the cost incurred to SaaS providers, eventually to reduce prices of cloud services to end users.

## 3. Problem Definition

Assume an enterprise that manages multiple datacenters (DC) located at different regions. Each location operates under the control of a local Regional Transmission Organization (RTO), so that electricity pricing varies from location to location and possibly also with time, depending on the specific policy in use. A total of *N* servers take part in running a common web application across *L* locations. In the notation that follows, we use superscripts to denote location and subscripts to indicate elements within a location. Each location has *n*^*i*^ servers, 1 < *i* < *L*. If $p_{j}^{i}(t)$ is the average power (in kW) consumed by server *j* at DC *i* and obtained at time *t* since a specific epoch *t*_0_, the average energy cost per hour is:

(1) $$E^{i}(t)=\sum_{j=1}^{n^{i}}p_{j}^{i}(t)\gamma^{i}(t),$$

where $\gamma^{i}(t)$ is the electricity price (rate) at DC *i* at *t* which remains constant during the last evaluation period (i.e., since the epoch *t*_0_ to *t*). The average power $p_{j}^{i}(t)$ may include pro rata the contribution of indirect sources, such as lighting, cooling, and other servers. The average energy cost per server and per hour related to running the web application and observed at time *t* is:

(2) $$x(t)=\frac{1}{N}\sum_{i=1}^{L}E^{i}(t).$$

While the general problem is to minimize the energy cost *x*(*t*), it is also crucial to consider that long request response times can have a detrimental impact on enterprise revenue. A simple example is an electronic commerce application where a slow system directly impacts transaction rates and, therefore, revenue. However, a similar rationale could be applied to other services. In addition to content quality, system responsiveness can affect the user perception of service quality and therefore impact the size of the user base over time. The exact definition of revenue loss due to response delay depends on application specifics and the needs of the enterprise. We formulate a basic expression for the purposes of our study assuming that revenue loss increases linearly with the average response time observed at each DC *i*:

(3) $$D^{i}(t)=\delta \;d^{i}(t),$$

where *δ* is the revenue loss factor and *d*^*i*^(*t*) is the average response time observed at location *i*. The average revenue loss of the system per server between the epoch and *t* is therefore:

(4) $$y(t)=\frac{1}{N}\sum_{i=1}^{L}D^{i}(t).$$

The combined cost is then described as:

(5) z(t)=x(t)+y(t).

The problem is how to achieve the optimal cost through routing web request to the *N* servers available. Iterative or direct methods may not be efficient enough to deal with the highly dynamic nature of this system where the electricity prices, energy demand and response time are all variable. We need a rapid decision-making system to make autonomous routing decisions based on the performance of the previous decisions in a dynamically changing environment. This provides us motivation to approach the problem with a learning method.

## 4. Routing with Learning Automata

**Figure 1.**
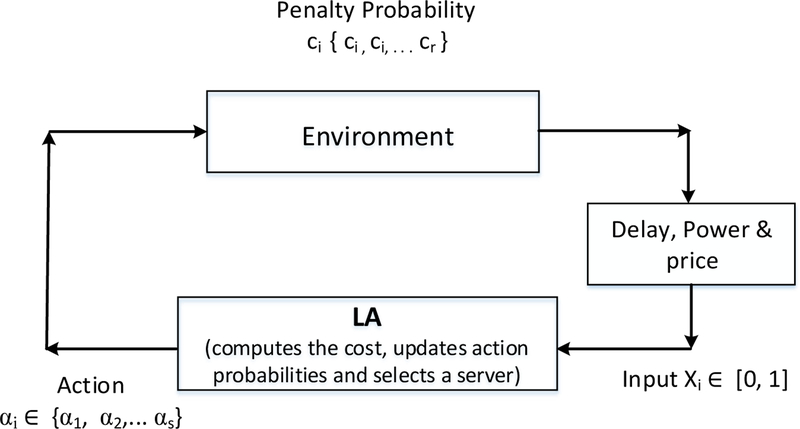
Stochastic learning automaton.

To better explain the proposed method, we first provide a brief overview of how Learning Automata can be applied to a routing problem. A Learning Automaton (LA) is an autonomous decision-making entity that learns to make decisions so as to bring optimal performance in the environment in which it is operating [25,26]. It implements a specific case of reinforcement learning [27]. An LA makes stochastic selection of an action from the list of available actions and observes its performance; if it sees favorable performance from the selected action, then it increments the selection probability of that action and adjusts the probabilities of other actions; if the selected action produces unfavorable response, then it penalizes the action by decreasing its selection probability. It starts with zero knowledge about the environment and learns on its own to select optimal actions. An action represents a server selection for handling a web request. An automaton is described by a quintuple {ϕ,s,X,G,A}, where *X* is the set of inputs i.e., X∈{0,1};ϕ is the set of internal states $\left\{\phi_{1},\phi_{2},\ldots ,\phi_{m}\right\}$ *s* is the output set $\left\{s_{1},s_{2},\ldots ,s_{n}\right\}$ with n≤m;G:ϕ→s denoting the output function and *A* is the algorithm that computes the state ϕ(t+1) from state ϕ(t) depending on the response *X* it received from the environment. Figure 1 depicts the interaction of LA with its environment.

The LA are classified as P-model, Q-model and S-model based on the responses it receives from the environment [25]. In P-model LA, the responses from the environment are mapped into binary values 0 and 1; in the Q-model, the responses are mapped into a finite number of values in the interval [0,^1^] and in the S-model, they are mapped into continuous values in the interval [0,^1^]. The LA are also classified based on the learning parameters used to update the selection probabilities.

1. **Reward Penalty (RP):** The learning parameter *b = a* and the selection probability of the server is rewarded or penalized equally when the selected server resulted in favorable or unfavorable performance.
2. **Reward Inaction (RI):** When *b* = 0, there will be no penalty for unfavorable response from the selected server (No action) and the server will be rewarded for favorable response.
3. **Reward Penalty-*ϵ* (RP-*ϵ*):** *b* << *a*, the selected action is penalized very little for unfavorable response.

The constants *a* and *b* are known as reward and penalty parameters, respectively.

The LA are broadly classified into fixed structure stochastic automata (FSSA) and variable structure stochastic automata (FSSA). In FSSA, the action probabilities are fixed i.e., transition probabilities are time invariant, whereas, in FSSA, the actions’ probabilities are updated based on the performance. The LA are classified as ergodic and non-ergodic according to their Markovian property. The ergodic LA does not lock itself into choosing any one action and it converges with probability distribution independent of initial distribution [28], whereas the non-ergodic LA has absorbing states and converges to an action which has minimum penalty probability with probability 1 [29]. The ergodic LA are suitable for non-stationary environments [28,30] and non-ergodic LA are apt for stationary environments. Both *RP* and *RP-ϵ* have ergodic Markovian properties and *RI* has non-ergodic properties. As many works from the literature illustrate the use of ergodic automata in network and communication applications [31–37], we are inspired to use the S-model Reward Penalty-*ϵ*, for making routing decisions in web applications. The servers operating in spatio-temporal electricity market and unpredictable web traffic patterns represent a non-stationary environment and justify the need for a VSSA with ergodic properties.

## 5. Cost-Aware S-Model Reward Penalty Epsilon (CA-S) Learning Automaton

A proxy server operates as a front end for a given web service. The service is handled by a set of origin servers and each server may be deployed at a different DC location. The proxy receives web requests from the users and forwards them to the origin servers following certain criteria. The reply from each origin server is sent to the proxy, which forwards the content to the client. The CA-S LA supporting the proxy’s decisions allows for achieving an adaptive and autonomous selection of servers based on observed costs that are mapped to selection probabilities.

Servers start with equal probability. When the LA receives a response from an origin server, it notes the request response time and the origin server’s power level to calculate a combined cost that serves to either increase (reward) or decrease (penalize) its selection probability. In the S-model LA, the actions are rewarded or penalized in a proportional amount to the performance. The amount of reward is larger for a higher favorable response and smaller for a lower favorable response. In the same way, the amount of penalty is larger for higher unfavorable performance and smaller for lower unfavorable response. The performance of the server is given by the combined cost of energy and revenue loss. The LA computes the normalized cost value of the current server to make the reward or penalty proportional. The calculation of the normalized cost and the reinforcement equations used to update the selection probabilities are explained next.

The combined cost *z*(*t*), as defined by Equation (5), is computed for all of the servers after each transaction, using the response time (delay) and energy cost as explained in Section 3. Even though the response is received from the selected server, the costs are updated for all server entries to accommodate the time varying electricity prices at each location. The exponential average cost *prev_cost* at an instance *t* is calculated from the current cost *z*(*t*) as follows:

(6) prev_cost(t)=α*z(t)+(1−α)*prev_cost(t−1),

where *α* is the weight given to the most recent value. The cost obtained from the selected server at a location (index) *v* at time *t* is normalized as *β*_*v*_(*t*):

(7) $$\beta_{v}(t)=\frac{\left(prev\_{\mathrm{cost}}_{v}(t)-m_{1}(t)\right)}{m_{2}(t)-m_{1}(t)},\;\text{where}\;m_{1}(t)=\min\left\{prev\_cost_{\text{1}}(t),\;prev\_cost_{2}(t),\cdots ,prev\_cost_{r}(t)\right\}\;\text{and}\;m_{2}(t)=\max\left\{prev\_cost_{1}(t),\;prev\_{cost}_{2}(t),\cdots ,prev\_cost_{r}(t)\right\}.$$

The normalized value *β*_*v*_ of *prev_cost* determines the amount of reward or penalty. If the selected server gives the minimum delay and minimum energy cost among all servers then *β*_*v*_ will be zero and it will receive a larger reward; if the selected server gives the largest *prev_cost*, it will be penalized more because *β*_*v*_ will be larger. The selection probabilities of the servers are updated as follows:

(8) $$P_{i}(t+1)=P_{i}(t)+a(1-\beta )\left(1-P_{i}(t)\right)-b\beta \;P_{i}(t),\;for\;i=v,\;P_{i}(t+1)=P_{i}(t)-a(1-\beta )P_{i}(t)+b\beta \left(\frac{1}{N-1}-P_{i}(t)\right),\;\forall i\neq v.$$

**Figure 2.**
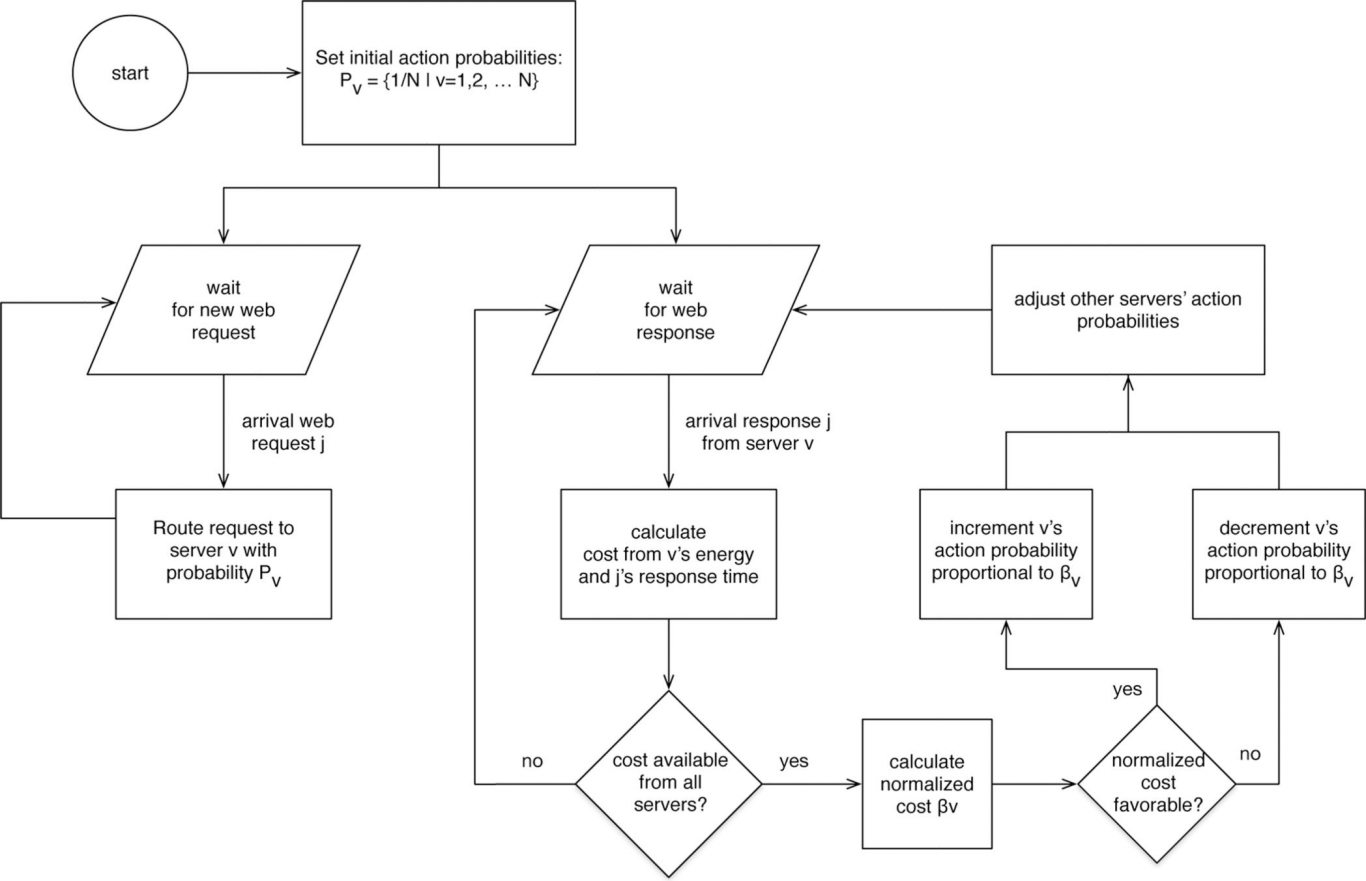
Flow chart of the proposed algorithm for dynamic web request routing with the Cost-Aware S-Model Reward Penalty Epsilon (CA-S) automaton.

The constants *a* and *b* are the learning parameters. The use of *β*_*v*_ in reinforcement equations makes the learning rate adaptive to the servers’ responses. Figure 2 explains the action flow executed by the LA when making a server selection and the learning flow when a response arrives. Optionally, automaton decisions can be further modulated with a random walk process, which occurs with probability *P*_*rw*_, to reduce the risk of reaching a local minima.

## 6. Evaluation of Parameter Selection Using Simulation

The performance of the proposed system may be affected by different learning parameters: reward factor *a*, penalty factor *b*, memory weight factor *α*, and random walk probability *P*_*rw*_. In addition, operational factors, such as the number of servers *N*, total traffic intensity *λ*, electricity rates (price) *γ*^*i*^ prevalent at each location *i* and the selected revenue loss factor *δ* determine the specific system behavior.

We use simulation to evaluate the impact of these factors. The advantage of the approach is that it provides a common and repeatable evaluation platform that allows for achieving high statistical accuracy. The simulation is event-driven and models the request generation as an exponential process with parameter *λ* (i.e., the total traffic intensity arriving at the system). The user requests are initially routed to a server (forward proxy server) that decides the actual origin server to handle each arriving request. The simulation keeps track of the communication dynamics of both requests and replies. Requests have a fixed length of 100 bytes. Reply lengths are exponentially distributed with an average size of 100 Kb.

The proxy is connected to the origin servers through connections of different delay. To simplify the simulation model, we assumed that a single server was used at each location. Delays from the proxy to the origin servers were distributed linearly from 10 ms to 1 s. The transmission rate of all the links was set to 2 Mbps. As a reference, the response time obtained with two or more servers is approximately 1.5 s. Each server has a single core and requests are served according to the first-come-first-serve model. We further assume that all the servers are of identical characteristics, consuming 100 W while idle and 150 W while transmitting or receiving data. Electricity prices (*γ*) were also linearly distributed among the different sites from 5 c/kWh to 15 c/kWh. The revenue loss factor was fixed at *δ* = 0.1 to produce comparable values for energy and delay costs.

### 6.1. Parameters a and b

**Figure 3.**
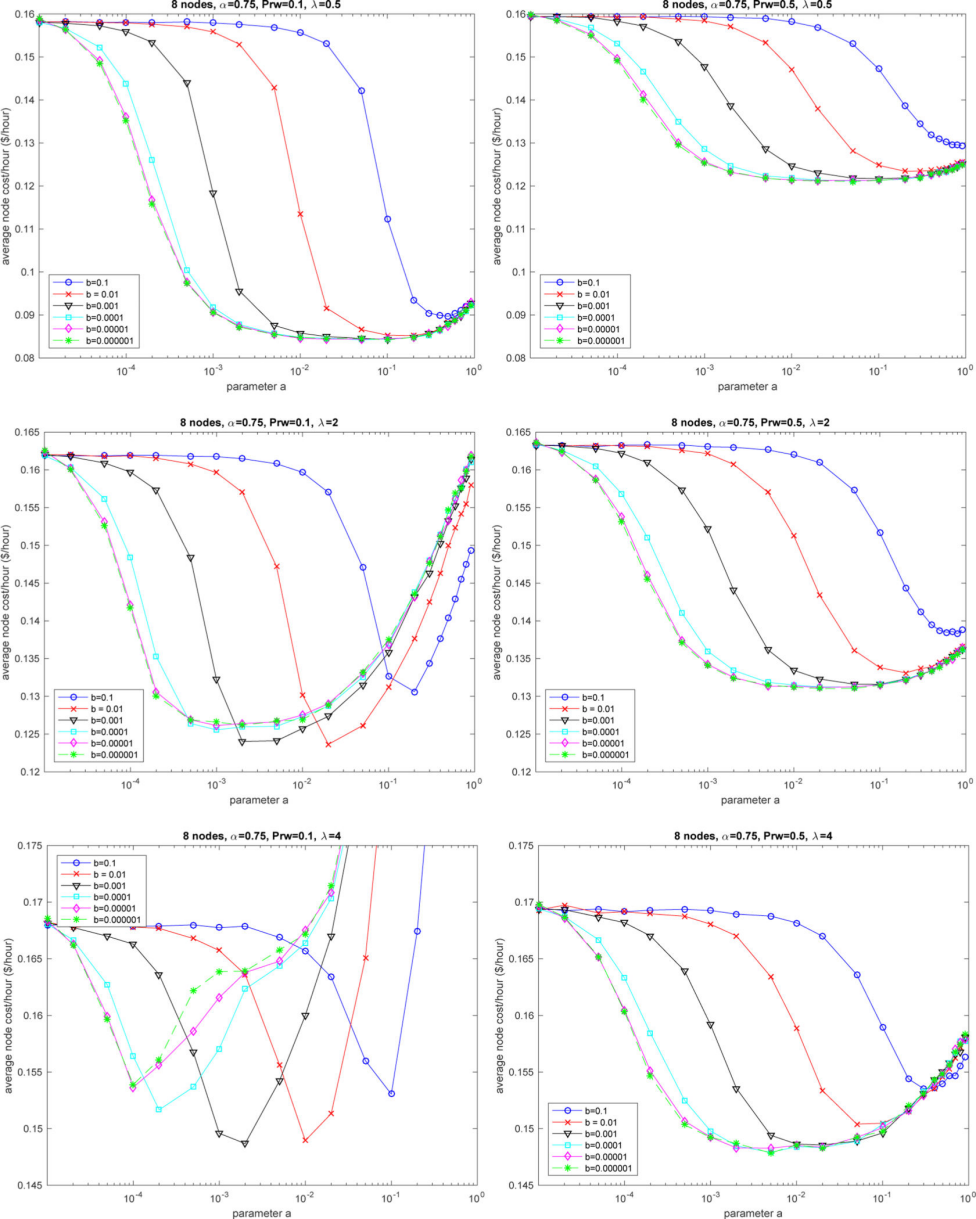
Impact of learning parameters *a* and *b*.

Figure 3 depicts the average cost per node and per hour for a 8-node system (8 locations) and memory factor *α* = 0.75 for different values of learning parameters *a* and *b*. The average cost is calculated by the simulator by averaging the observed costs of 100 runs each handling 30,000 requests. The standard deviation of the mean was very small in all cases, so we have opted for not including the confidence interval in the charts for visual clarity. The three plots on the left part of the figure depict the case with *P*_*rw*_ = 0.1. The three charts on the right correspond to the case *P*_*rw*_ = 0.5. We have used the same scale on the figures to better appreciate the differences. Each of the plot rows in the figure shows the results of different traffic intensity: low *λ* = 0.5, medium *λ* = 2, high *λ* = 4. The maximum utilization is in the range 5–6 req/s for the 8-node system that we considered.

The results show high parameter sensitivity for both *a* and *b*. The best values for *b* depend on the selected value for *a* and vice versa. We observed good performance with values for *a* around 0.01 with detrimental effects both by using smaller or greater values. With *a* = 0.01, we observed that *b* = 0.001 provided reasonable low cost for different values of *λ*. On the other hand, increasing the chances of random walk (right-hand side plots) did not produce major improvements. It is interesting to note that the optimization opportunities reduce with traffic intensity. With the lowest traffic rate, the cost range was around 8 c, whereas, with the highest traffic rate, the rate was about 2 c. Increasing the chances of random walk also reduce the cost range. For example, increasing *P*_*rw*_ to be near 1 for the low traffic case removes the optimization opportunities achieving the maximum cost of 0.16 for any value of *a* and *b*.

### 6.2. Parameter P_rw_

**Figure 4.**
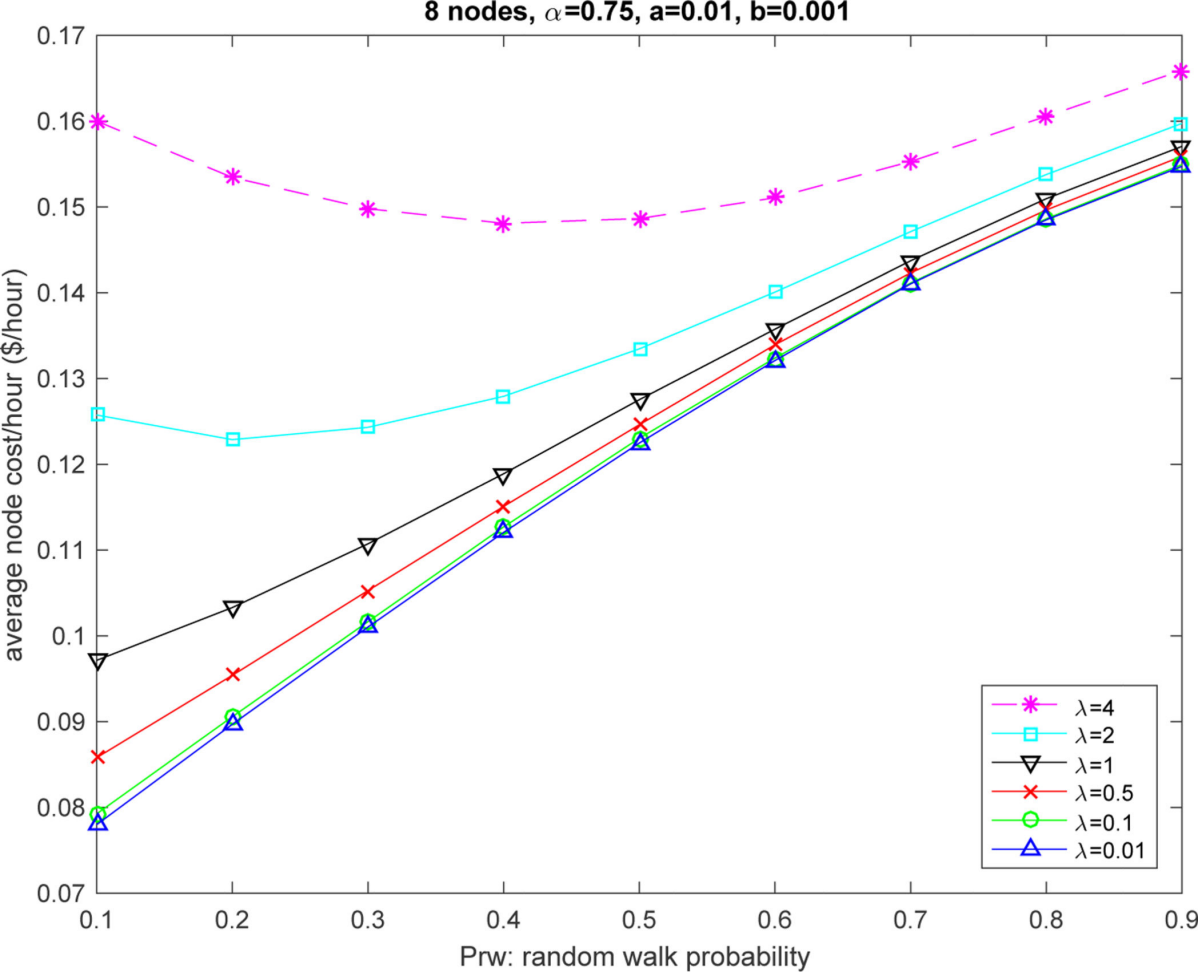
Impact of pure random decisions *P*_*rw*_.

Figure 4 provides further evidence of the impact of the use of pure random decisions in the system. We can observe that the cost impact of *P*_*rw*_ is minimal for medium traffic. For low traffic, we observe that the system is better off by not using pure random decisions. However, for high traffic, small values of *P*_*rw*_ may bring some performance improvement. Considering that the automaton can only be effective by receiving proper feedback, we note that, with the delays caused by high traffic congestion, the use of pure random actions can provide benefits over a regular automata with stale information.

### 6.3. Parameter α

**Figure 5.**
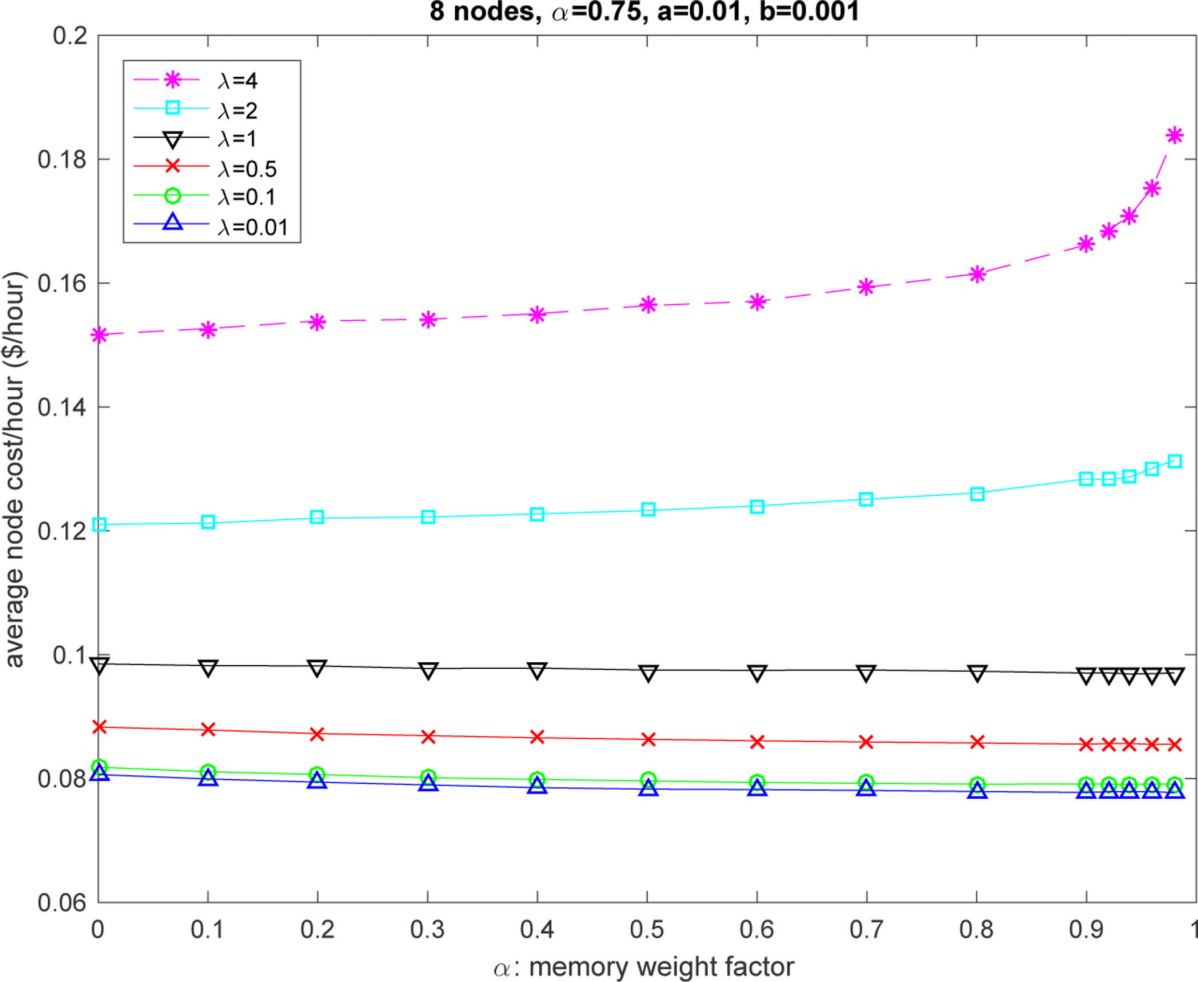
Impact of memory weight parameter *α*.

The memory weight *α* has little effect under low to medium traffic intensity (see Figure 5). For high traffic intensity, decisions are better off by using smaller weights, in other words, it is better to consider the latest cost rather than the cost average to modify the action selection probabilities as the information contained in the average can quickly become stale under congestion.

### 6.4. Number of Servers

**Figure 6.**
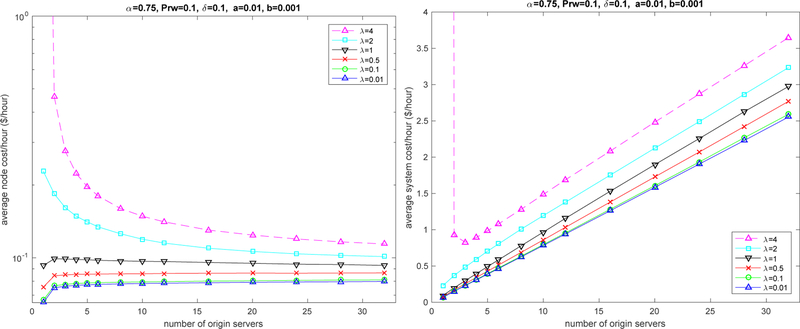
Cost impact of the number of servers; per node hourly cost (**left**); system hourly cost (**right**).

Figure 6 depicts the average node cost per hour as a function of the number of servers. Increasing the number of servers benefits the cost metric because, under the same traffic intensity, each server handles a lower request rate, therefore requiring less energy for the evaluation period). Certainly, adding servers does increase the system cost as depicted on the right-hand chart of Figure 6.

## 7. Testbed Evaluation

**Figure 7.**
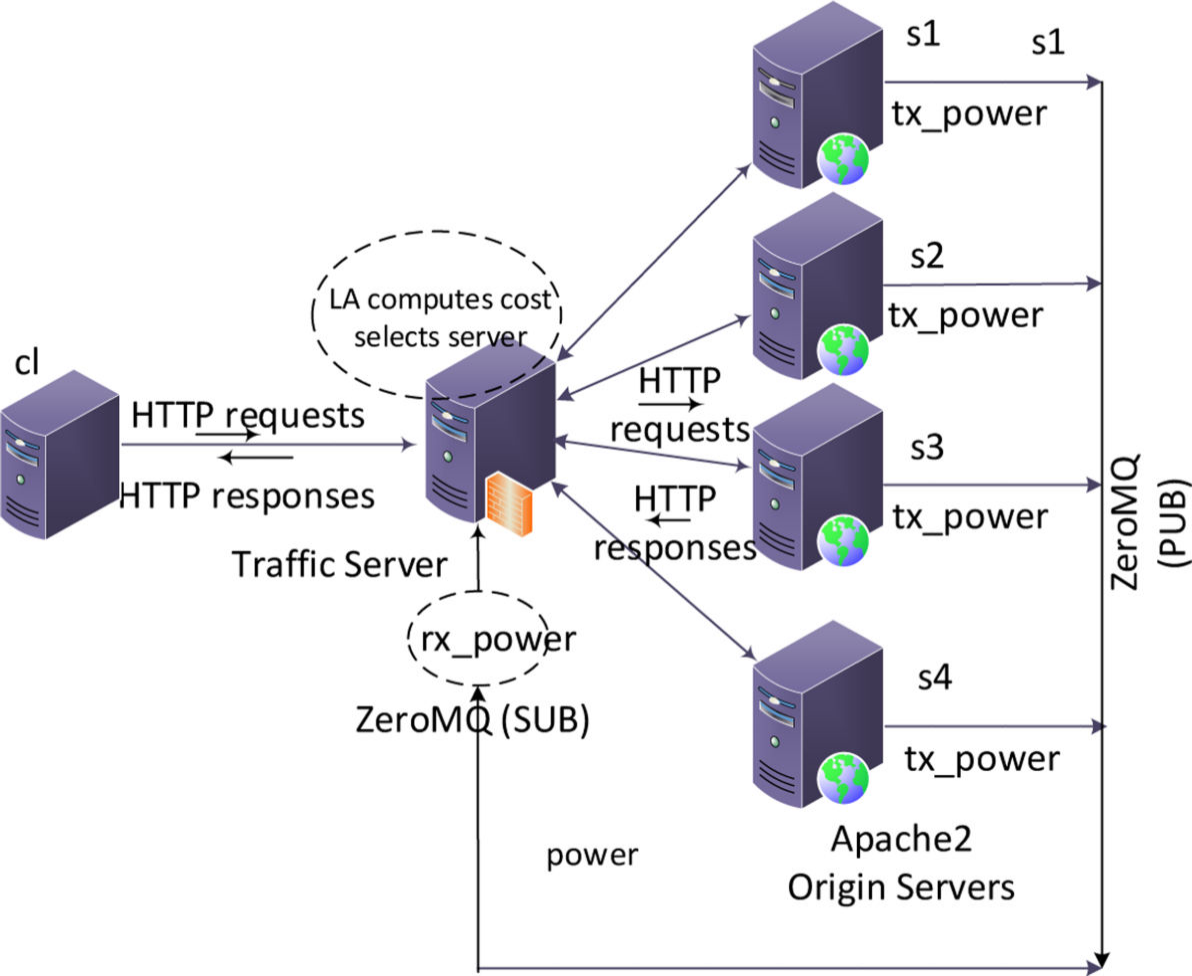
Experimental topology deployed in CloudLab.

We have conducted experiments on CloudLab [6]. The topology consists of six servers of type 220g1 as shown in Figure 7. All servers are equipped with two Intel E5–2630 v3 8-core central processing units (CPUs) at 2.40 GHz (Haswell EM64T), 128 GB error-correcting code memory (ECC), a dual-port Intel X520-DA2 10 Gbps network interface card (NIC) and two 1.2 TB disks. All machines run Linux Ubuntu 16.04. Apache2 web server was installed on the nodes s1, s2, s3, s4 to operate as origin servers. All of the nodes provide power monitoring capabilities. We have obtained the power usage of origin servers using the Intelligent Platform Management Interface Tool (*ipmitool*). Apache Traffic Server 7.1.2 was installed on the node *proxy,* which serves as a reverse proxy server between client and the origin servers. To emulate Internet (wide area network) delays, we introduced the following random artificial delays to the links connecting origin servers to proxy using Network Emulator (Netem): 10.0 ± 2.0, 0 (no delay), 20.0 ± 5.0, and 5.0 ± 2.0 ms, respectively.

We implemented our method for web request routing (CA-S) as a policy function with the balancer plug-in of the traffic server. The LA implemented as the policy function will measure the delay in serving responses, read the power utilization of servers and compute the total cost after each transaction. It then updates the selection probabilities of the servers and selects a server at random for the next incoming request. An application (*tx_power*) written in C++ at each origin server queries the power usage from *ipmitool* at one-second intervals and sends the power values in watts to both *proxy* and the client node *cl* using *PUB/SUB* messaging service of ZeroMQ. An application written in C++ (*rx_power*) running on the *proxy* node subscribes to all the origin nodes and receives the power values. It then writes these values into a memory shared with the traffic server. The PUB/SUB messages are transmitted out of band with the HTTP requests. A python application running from the client node *cl* sends HTTP requests for static files of various sizes to the origin servers via *proxy* node in Poisson distribution. The requests are sent from 20 requests/s to 300 requests/s in steps of 20 requests/s. Each experiment runs for a duration of 10 min and was repeated several times. The service times and power utilization are recorded at the client node for plotting the results.

The probabilities are updated after each transaction based on the performance of servers when each of them has been selected for a transaction. The server which serves the requests with the minimum combined cost will have the highest probability in the long run. The server having the highest probability will have the most number of chances to serve the requests. However, once its performance starts deteriorating, it will not hold the highest probability and the probability of the server having the next best performance starts increasing. As the LA used in this work possesses ergodic Markovian properties, it does not lock itself into any one server, and optimal performance could be achieved in spite of dynamic changes in the electricity prices and with the variations in response times. To better illustrate the roles of the raw electricity costs and revenue loss due to high response times, we have introduced an additional parameter *k* into the objective function: *z*(*t*) = *kx*(*t*) + (1 − *k*)*y*(*t*). Depending on the value selected for *k*, the modified goal function simulates the use of different electricity rates (keeping *γ* fixed) or different choices for *δ*.

## 8. Results

We have conducted several experiments to test the sensitivity of the algorithm to some of the parameters used in the algorithm. Unlike the simulation, the CloudLab environment offers realism, but also some limitations—for example, the current method employed for energy monitoring requires the implementation of a process that adds overhead to the servers. We have tested the performance of our proposed approach using two price scenarios, with two values of *k* (0.2 and 0.9), two values of *α* (0.1 and 0.9) and different values for the penalty parameter *b*.

### 8.1. Baseline Methods

We consider the round robin (*RR*) and the minimum cost flow (MC) server selection methods to produce baseline performance. The *RR* is a static policy in which servers are selected in turn and each server handles an equal number of requests. It is a widely used method for load balancing and request forwarding, in particular, when servers are homogeneous and workload is uniform [38–42]. It is commonly used in the web industry because of its low complexity.

A second baseline method is adopted from [11]. In that work, the optimization of electricity costs for a distributed DC system in a multi-electricity market was handled by mixed integer linear programming. The optimization problem was converted into the minimum cost flow problem given the challenges involved. Brenner’s algorithm was used to find the minimum cost flow for a network of five front-end web portal servers connected to Google’s data centers in three locations. In that work, the front-end web servers act as sinks and the DCs act as sources with the links that connect them carrying the electricity costs. The process consists of the following steps. First, the amount of workload for each data center is computed based on the link costs (electricity price). Second, the required number of servers to be provisioned is computed from the assigned workload for each DC by considering the delay and the number of active servers as constraints. Our MC implementation has no constraints. We find the minimum cost flow of a network of four data centers connected to one front-end server (proxy) through links carrying a combined cost of energy and revenue loss (delay). The combined cost is computed after each transaction as described in Equation (5), and the server having the least cost among all servers is chosen greedily for the next incoming request.

In the proposed CA-S algorithm, the LA observes the current electricity price rates, power consumption of all servers, and response time from the selected server (for each transaction). It then updates the selection probability of the current server proportionally to its performance by comparing its cost to the costs of other servers through the learning process. To make a new server selection, it makes a stochastic selection. By updating the selection probabilities based on the observed performance, it achieves a server selection that is adaptive to the environment. The amount of reward or penalty to the selected server is based on how far the cost is from the minimum and maximum observed costs. Thus, by continuously tracking the state of the environment, the LA makes adaptive decisions and approaches optimally.

Even though the MC method measures the costs in a similar way to CA-S, i.e., after each transaction, it may continue selecting the same server. For example, at certain time, the best server may respond with higher delay than usual because of the nature of a given request (e.g., the query may require the execution of a certain complex computation or a large data transfer). In that case, MC may move to a non-optimal server and will continue to send requests to that server until the cost exceeds the cost of the unusual request. In the long run, MC will produce suboptimal performance.

### 8.2. Impact of the Offered Load and the Price Scenario

Table 1 shows the electricity price rates for each of the four servers used in our tests under each of the two scenarios. The rates are given in cents per kWh. The selected values correspond to typical average electricity rates that are currently in use in different states in the U.S [43].

**Figure 8.**
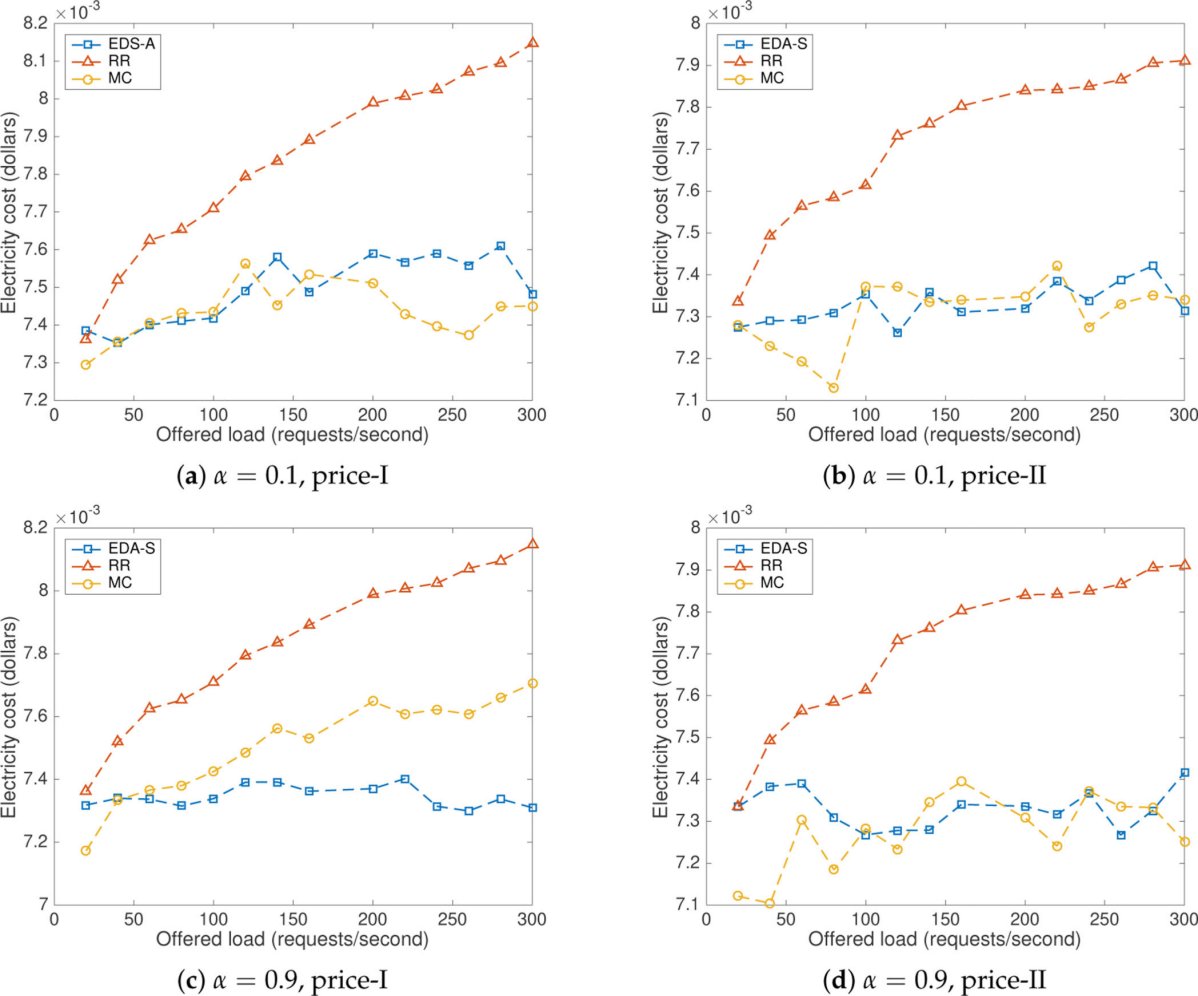
Electricity cost with CA-S tested in CloudLab.

**Figure 9.**
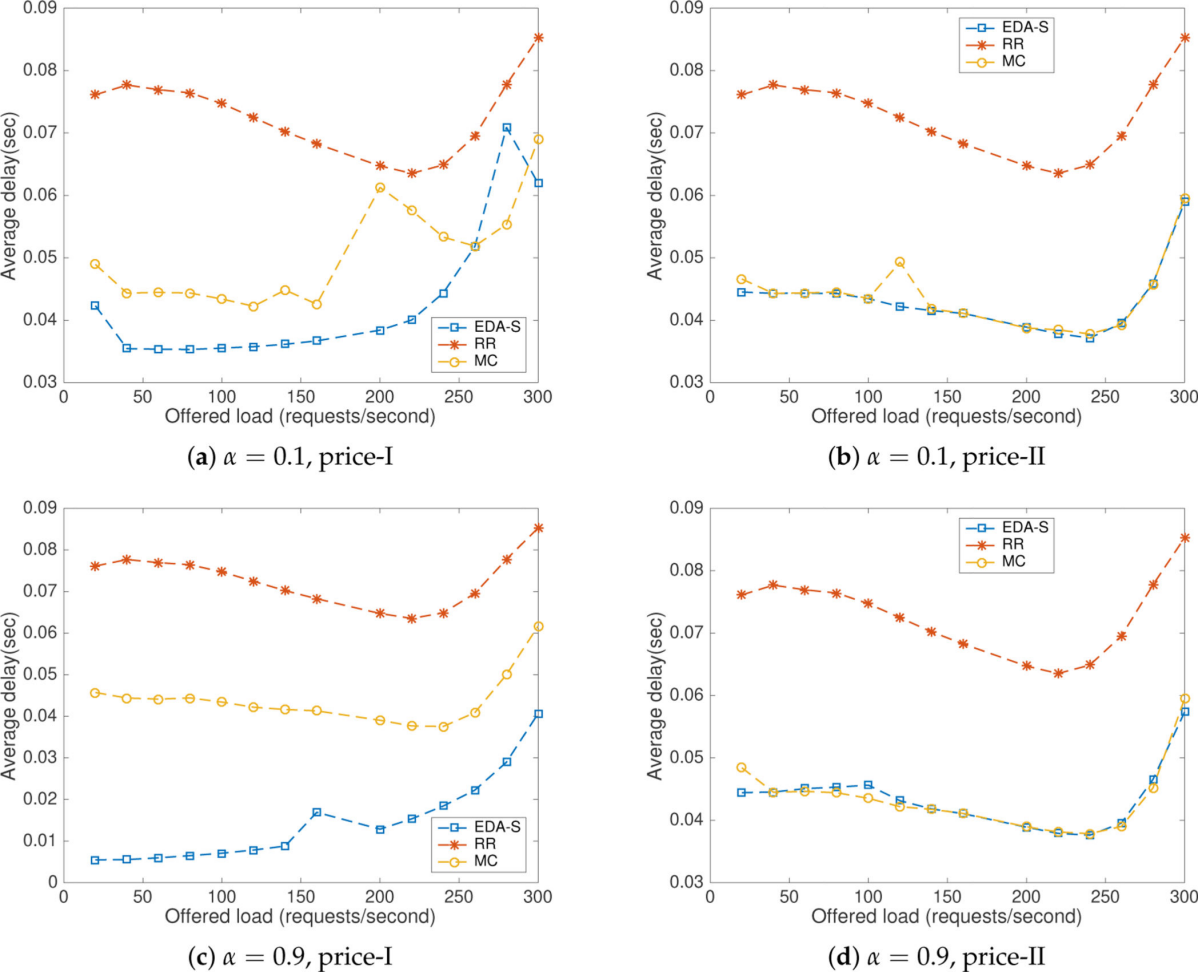
Average delay with CA-S tested in CloudLab.

In price scenario-I, servers *s*1 and *s*2 are located in regions with the lowest electricity prices. As server *s*2 lack any configured delay, it is commonly the optimal server, at least under low load, with the selected values. In contrast, in price scenario-II, servers *s*3 and *s*4 have lower prices than the first two servers. Server *s*4 is configured with lower delay than *s*3 and hence it is the optimal server for most requests generated in this scenario. Measurements for the total average electricity cost and the average delay are plotted in Figures 8 and 9 for *k* = 0.9 (i.e., revenue losses due to high response times are given higher importance than raw energy costs). Figure 8a,b shows that the proposed approach performed similar to the MC method and outperformed *RR* in both price scenarios with *α* = 0.1. Figure 8c,d shows that CA-S has out performed both *RR* and MC in price scenario-I and performed similarly to MC and better than *RR* in price scenario-II for *α* = 0.9.

From Figure 9a–d, it is clear that CA-S has outperformed both *RR* and MC with lower average delays for both values of *α* in price scenario-I and performed similar to MC or better than *RR* in price scenario-II. In price scenario-I, as the fastest server also has the lower price, the average delay was optimal. In price scenario-II, server *s*4 has the lowest price with a configured delay of 5.0 ms ± 2.0 ms. The proposed method selects *s*4 most of the time when attempting to optimize the combined cost, producing an increase on the average delay. We have verified the number of requests serviced by each server in both cases using traffic server logs.

**Figure 10.**
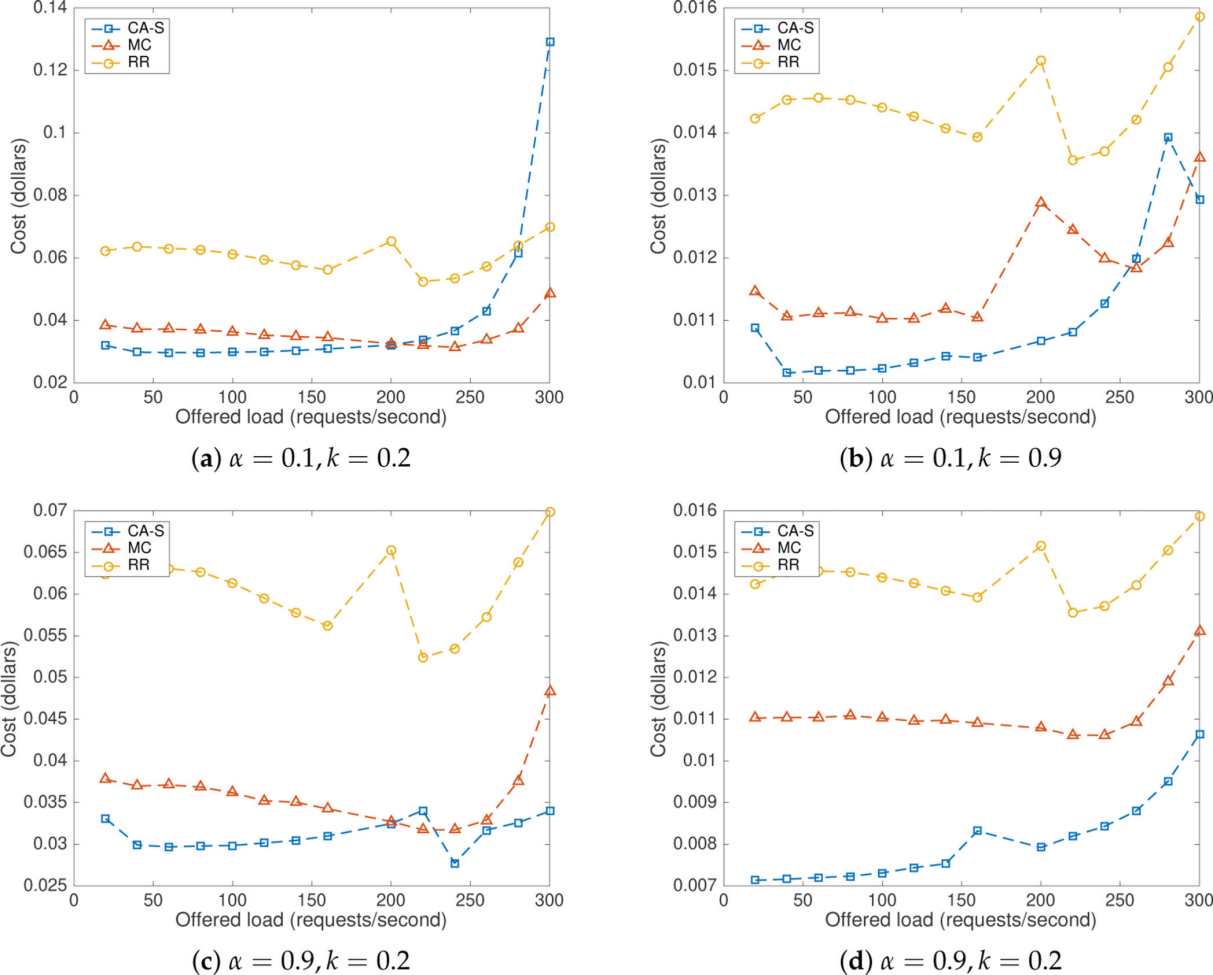
Combined cost with CA-S in price scenario-I.

Figure 10 depicts the combined cost incurred by the different algorithms used in this study under price scenario-I. The proposed CA-S method performed better than RR for both values of *α* and both values of *k*, except for high values of *λ* (see Figure 10a). In this price scenario, the CA-S method performed generally better than MC for *λ* values up to approximately 200 req/s. When *λ* = 0.9 and *k* = 0.9, the CA-S method produced better costs than MC. When giving higher importance to the current cost (i.e., *α* = 0.9), the most recent variations of both energy and delay costs are better tracked and the rewards and penalties update the action probabilities faster. Thus, higher values of *α* makes the selection better adaptive, which yields lower costs. These results are consistent with our observations obtained in simulation for low to medium traffic rates. We also observed that a high value of *k* in price scenario-I produced lower costs.

**Figure 11.**
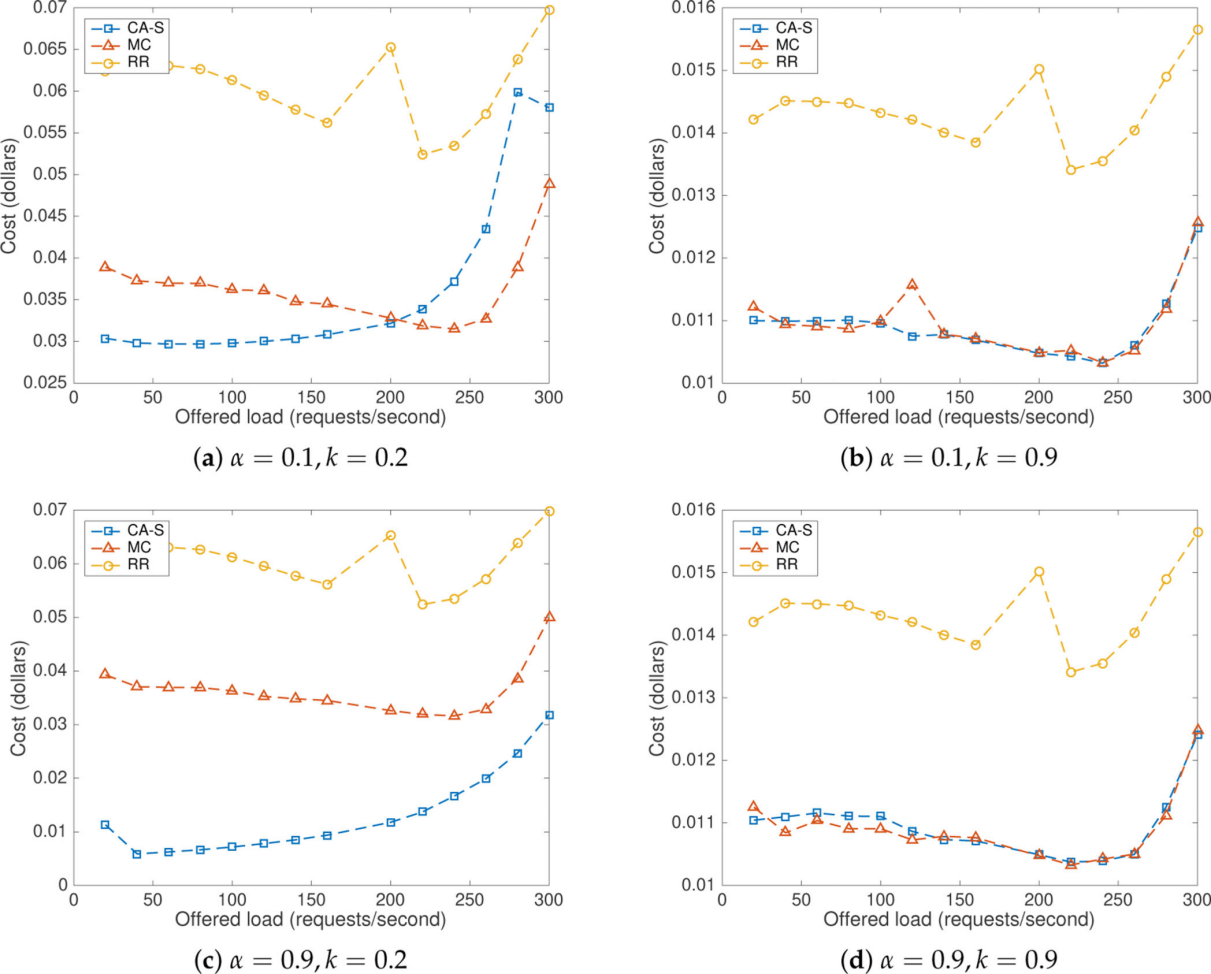
Combined cost with CA-S in price scenario-II.

Figure 11 describes the combined cost incurred by using all of the three algorithms under price scenario-II. The CA-S method performed better than RR for both values of *α* and for both values of *k*. Again, CA-S performed better than MC for *λ* values up to 200, but, after that point, performance decreases as shown in Figure 11a. As indicated by Figure 11b,d, CA-S performed about the same as the MC method. The high value of *k* helps the LA to optimize energy costs with more importance than delay. Server *s*4 was therefore selected more frequently. As *s*4 has an artificial delay associated with it, the combined cost increased and hence performance decreased. As shown in Figure 11c, it shows superior performance than MC. A small value of *k* (0.2) reduces the importance given to energy cost increasing the selection probability of *s*2 and *s*4. In addition, a high value of *α* helps to update the selection probabilities.

The Minimum Cost flow algorithm (MC) performed better than RR in both price scenarios and equal to CA-S in price scenario-II for *k* = 0.9 in both *alpha* values. This is because MC selects a server based on the performance similar to CA-S by using the feedback mechanism. However, it does not have a learning ability and no stochastic selection. When MC finds a server with minimum cost, it tries to send every request to the same server until the cost of the server becomes larger than the other server. As the performance of the other servers is unknown, MC tries to compare the cost of the current server to stale values. The approach works well under stationary conditions, where all servers produce about the same performance. However, when energy costs and delays can dynamically vary, the MC method underperforms.

RR is a common baseline method in the literature that, because of its low overhead, is commonly used for real-time request forwarding. However, we experimentally observed that it produces suboptimal performance in general due to its lack of dynamism. On the other hand, this method would work well with stationary assumptions with servers offering similar capabilities.

### 8.3. Parameter b

**Figure 12.**
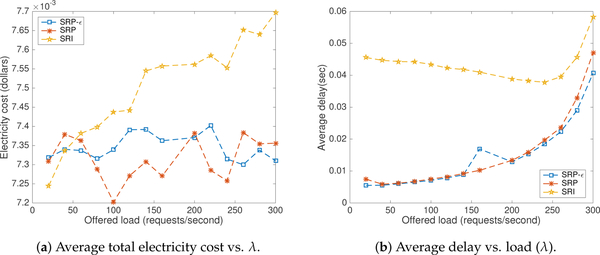
Influence of penalty parameter *b*.

Figure 12 shows experimental evidence of the performance of CA-S using different choices for the learning automaton (see Section 4): *SRP* (*b* = *a* = 0.078), *SRI* (*b* = 0), and *SRP-ϵ* (*b* << *a*, *a* = 0.078, *b* = 0.006). The results suggest that *SRP* is the best choice given its performance compared to the other two models.

## 9. Conclusions

We have proposed a dynamic method for web request routing among replicated and geographically distributed servers that operate under the authority of different electricity markets. The Cost Aware S-model Reward Penalty Epsilon (CA-S) method effectively exploits the spatial-temporal price and performance differences of the servers available to reduce the average total cost as compared to conventional methods. The proposed CA-S method continuously balances energy costs and revenue losses that are associated with high-latency web responses when routing web requests. We have reported on the sensitivity of the CA-S to the various parameters used by the algorithm through both simulation and experimentation in CloudLab. We considered the two test scenarios. In one scenario, the server with the lowest delay had the lowest electricity cost rate. The opposite case was represented in the second scenario. Our results indicated that CA-S provides in general better performance than related methods (round robin and minimum cost flow) regardless of the test scenario. Our future work explores the issues related to the use of multiple proxies at distributed locations to remove the possibility of a single point of failure.

## Figures and Tables

**Table 1.** Price scenarios (given in cents per kWh).

Scenario	*s*1	*s*2	*s*3	*s*4

*I*	4.70	4.8	14.47	13.75
*II*	13.75	14.47	4.8	4.7
